# Pregnant women’s experiences and perceptions of participating in the EVERREST prospective study; a qualitative study

**DOI:** 10.1186/s12884-019-2277-8

**Published:** 2019-04-30

**Authors:** Merryl E. Harvey, Anna L. David, Jade Dyer, Rebecca Spencer

**Affiliations:** 1 Faculty of Health, Education and Life Sciences, Birmingham City University, City South Campus, Westbourne Road, Edgbaston, Birmingham, B15 3TN UK; 2 EGA Institute for Women’s Health, University College London, NIHR University College London Hospitals Biomedical Research Centre, Maple House, 149 Tottenham Court Road, London, W1T 7DN UK; 3 Comprehensive Clinical Trials Unit, Institute of Clinical Trials & Methodology, University College London, 90 High Holborn, London, WC1V 6LJ UK; 4 EGA Institute for Women’s Health, University College London, 86-98 Chenies Mews, London, WC1E 6HX UK

**Keywords:** Pregnancy, Fetal growth restriction, Placental insufficiency, Women, Interview, Qualitative, Experience

## Abstract

**Background:**

The EVERREST Prospective Study is a multicentre observational cohort study of pregnancies affected by severe early-onset fetal growth restriction. The study recruits women with singleton pregnancies where the estimated fetal weight is less than the 3rd centile and below 600 g, between 20 + 0 and 26 + 6 weeks of pregnancy, in the absence of a known chromosomal, structural or infective cause.

**Method:**

The reported study was retrospective descriptive qualitative interview study of women who had participated in the EVERREST Prospective Study. The aim of this study was to explore the experiences and perceptions of pregnant women taking part in research during a pregnancy affected by severe early-onset fetal growth restriction. Audio-recorded semi-structured telephone interviews were conducted with a purposive sample of 12 women, at least 1 year after delivery of their baby. Two of these pregnancies had ended in stillbirth and one in neonatal death, reflecting the outcomes seen in the EVERREST Prospective Study. Participants gave informed consent, were 16 years or older and were interviewed in English. A topic guide was used to ensure a consistent approach. Questions focused on pregnancy experiences, involvement with the EVERREST study and potential involvement in future research. Recordings were transcribed verbatim for thematic analysis using NVivo10.

**Results:**

Four broad themes were identified; ‘before joining the EVERREST Prospective Study’, ‘participating in research’, ‘information and support’ and ‘looking back and looking forwards’. Each broad theme incorporated several subthemes. All participants recalled their reaction to being told their baby was smaller than expected. The way this news was given had a lasting impact. A range of benefits of participation in the EVERREST Prospective Study were described and the participants were positive about the way it was conducted. As a consequence, they were receptive to participating in future research. However, the findings suggest that research teams should be sensitive when approaching families at a difficult time or when they are already participating in other research.

**Conclusions:**

This study highlights the willingness of pregnant women to participate in research and identifies strategies for researchers to engage participants.

**Electronic supplementary material:**

The online version of this article (10.1186/s12884-019-2277-8) contains supplementary material, which is available to authorized users.

## Background

Fetal growth restriction (FGR) affects 8% of pregnancies and accounts for 30% of stillbirths [1]. Severe early-onset FGR occurs before 28 weeks gestation and is most commonly due to insufficient utero-placental blood flow termed placental insufficiency. As no treatment is currently available to improve fetal growth, management is delivery of the baby before intrauterine death or permanent organ damage [2]. A potential treatment for FGR is maternal gene therapy which aims to increase the local availability of Vascular Endothelial Growth Factor (VEGF) in the maternal uteroplacental circulation. Preclinical studies have shown that maternal uterine artery administration of adenovirus vectors containing the VEGF gene in pregnancy increases uterine blood flow, angiogenesis and fetal growth [3–6].

The EVERREST Project intends to perform a phase I/IIa trial of maternal VEGF gene therapy for severe early-onset FGR [7]. As the primary outcome will be to establish the safety of the intervention, it will aim to recruit women for whom the risks of fetal of neonatal death are high enough to justify potential risks [8]. These women will therefore face the psychological and emotional impact of the diagnosis and prognosis as well as potential clinical trial participation. The EVERREST Clinical Trial will be a dose-escalation trial and will not include a placebo arm.

It is fundamental to the ethical conduct of medical research that the potential benefits of a trial must be weighed against the risks and burdens for the participant [9, 10]. This is more than just the physical risks of any procedure or medication, and may include the burden of time taken to participate or the emotional impact of taking part in the research. When weighing these risks, researchers and Research Ethics Committees use a combination of “common sense, clinical judgement, prior experience, imagined personal substitution with the participant, and multidisciplinary consultation” [11]. These strategies have the potential to either over- or underestimate the burdens a trial places on participants, depending on the opinions and assumptions of those involved. This could be particularly relevant to the EVERREST Clinical Trial, where the clinical situation already places practical and emotional burdens on women.

In recent years increasing attention has been paid to women’s experiences of participating in research during pregnancy [12–17]. Many of the findings of these studies can be generalised to wider obstetric research, however some of their findings will inherently be specific to the trial or study in which the women participated. Furthermore, while many studies have used a qualitative approach to explore women’s experiences of pregnancy, including high risk and complicated pregnancies [18–22], we were unable to identify any studies that had addressed the experience of women with pregnancies complicated by FGR. In previous work by the EVERREST Consortium we had explored the attitudes of women and couples with previous pregnancies affected by severe early-onset FGR towards research in pregnancy [23]. We now wanted to investigate the experience of women with pregnancies affected by severe early-onset FGR who had taken part in clinical research.

In order to provide comparison data on untreated pregnancies for the future EVERREST Clinical Trial, the EVERREST Prospective Study was launched in March 2014 [7]. This recruits women with singleton pregnancies where the estimated fetal weight (EFW) is below the 3rd centile and below 600 g, between 20 + 0 and 26 + 6 weeks of pregnancy, in the absence of a known chromosomal, structural or infective cause. This is consistent with the recent Delphi consensus on the definition of early placental FGR [24]. Clinical data and biological samples are collected from participants but they do not receive any interventions.

As an extension to the EVERREST Prospective Study, semi-structured qualitative interviews were undertaken with women who had taken part in the UK arm of the study, to provide insight into the experiences of women with severe early-onset FGR who have taken part in research during pregnancy. This qualitative study also aimed to gain insight to the likelihood of mothers agreeing to participate in the EVERREST Clinical Trial. Qualitative telephone interviews have been used in a range of studies including those involving challenging and sensitive topics such as cancer, eating disorders, premature birth and post-natal depression [25–27].

## Methods

### Study aims

- To explore the experiences of women who took part in the EVERREST Prospective Study of experiencing a pregnancy complicated by severe early-onset fetal growth restriction
- To explore the experiences of women who took part in the EVERREST Prospective Study of taking part in a research study during pregnancy
- To provide insight to the likelihood of pregnant women agreeing to participate in the EVERREST Clinical Trial

This was a retrospective descriptive qualitative interview study of women who had participated in the EVERREST Prospective Study at University College London Hospital [7]. Audio-recorded, semi-structured, qualitative telephone interviews were conducted using a topic guide (MH). The study inclusion criteria were women who had taken part in the EVERREST Prospective Study in the UK, who had delivered their baby 1 year or more ago, were able to give informed consent, were aged 16 years or older and were able to take part in a telephone interview in English.

Of the 55 UK EVERREST Prospective Study participants who were eligible to take part in the interview study, 35 were either undergoing continuing follow-up or had given consent to receive information by post from the study team. Twenty-eight women were sent invitation letters, including a participant information sheet and consent form. A further seven women were given information about the interview study when attending the hospital for follow-up.

With participant consent the interviews were audio recorded to facilitate transcription and data analysis. The topic guide (Additional file 1) consisted of key and follow-up questions. The use of follow-up questions was determined by the participant’s response to the initial key question. The overall format of each interview was therefore slightly different. However, using the topic guide ensured that all key issues were covered.

Following the interview, the participants were sent a £20.00 shopping voucher to acknowledge their time. The recordings were transcribed by a professional secretarial service. The transcripts were then checked for accuracy (MH). Thematic analysis was undertaken using the software package NVivo 10 (MH). The transcripts were read to ensure familiarity with the content and context. Sections of the text were then coded. New codes were created when the data appeared to capture something different. The codes were then formed into broad themes and subthemes with each broad theme containing a number of subthemes. Once all of the transcripts had been coded, the coding framework was reviewed and amended. As a consequence, some of the original codes and sub-themes were combined, whilst others were separated until the final framework of broad themes and sub-themes was established [28]. We also examined the responses in relation to participant characteristics to investigate whether certain opinions or experiences were more common in subgroups of respondents. In accordance with accepted qualitative methods, data collection, transcription and data analysis was carried out concurrently [29–31] until data saturation was reached.

Approval for the study was obtained from London Stanmore Research Ethics Committee (13/LD/1254). It was possible that some women would find describing their experiences upsetting. Consequently, the purpose of the interviews was clearly identified on the participant information sheet and reiterated when the interview was arranged. Guidance on where to seek ongoing support was provided. Some women may have felt obliged to make positive responses, particularly about being part of the EVERREST Prospective Study. However, they were all reassured that their responses would be anonymised and it was anticipated that as the interviewer (MH) was not part of the original research team that participants would feel comfortable making their responses.

## Results

Participant recruitment took place between October 2016 and July 2017. Of nineteen women who indicated that they were interested in taking part, five could not subsequently be contacted. Interview arrangements were made with two potential participants who requested that the interview was rescheduled and then could not be contacted. Consequently, a purposive sample of 12 women was interviewed (Table 1). This sample size reflects that used in qualitative research where the intention is to provide insight to a range of participant experiences rather than generalise the findings to a wider population [32]. Sample configuration compared to that of all participants of the EVERREST Prospective Study who were eligible to participate in the interview study is described in Table 2.

**Table 1:** Participant information

Study number	Age bracket (years)	Parity	Birth weight (g)	Birthweight centile [50]	Mode of delivery	Gestation at delivery (weeks + days)	Baby outcome
01	40–45	G1 P0	460	2nd	CS	25^+ 0^/40	3 months total in 2 NNUs, now well
02	35–40	G4 P2^+ 1^	714	2nd	CS	27^+ 4^/40	Died 9 days old
03	30–35	G1 P0	1040	Between 0.4th and 2nd	CS	31^+ 3^/40	2 months total in 2 NNUs, now well
04	30–35	G1 P0	563	Between 0.4th and 2nd	CS	27^+ 5^/40	3 months NNU, now well
05	35–40	G1 P0	1020	Between 0.4th and 2nd	CS	31^+ 5^/40	2 months total in 2 NNUs, now well
06	35–40	G5 P2^+ 2^	2030	Between 0.4th and 2nd	Vaginal	37^+ 3^/40	Well
07	30–35	G1 P0	550	Below 0.4th	CS	28^+ 6^/40	3 months total in 2 NNUs, now well
08	35–40	G1 P0	2440	Between 2nd and 9th	Vaginal	38^+ 1^/40	Well
09	35–40	G1 P0	1190	Below 0.4th	CS	34^+ 6^/40	1 month in NNU, now well
10	30–35	G2 P1^+ 0^	1053	Between 2nd and 9th	CS	31^+ 2^/40	2 months total in 2 NNUs and 1 children’s hospital, now well
11	35–40	G5 P3^+ 1^	328	Below 0.4th	Vaginal	25^+ 0^/40	Stillbirth
12	35–40	G2 P1^+ 0^	472	Below 0.4th	Vaginal	28^+ 3^/40	Stillbirth

*G* gravida, *P* para, *CS* caesarean section, *NNU* neonatal unit

**Table 2:** Comparison of participants in this interview study and eligible UK EVERREST Prospective Study participants

		Interview study participants (*n* = 12)	UK EVERREST Prospective Study participants (*n* = 55)
Age (years)		36.5 (33.5 to 38)	34 (30 to 37.75)
Primigravida		7 (58%)	26 (47%)
Ethnicity:	White	7 (58%)	32 (58%)
	Asian	2 (17%)	11 (20%)
	Black	2 (17%)	11 (20%)
	Other	1 (8%)	1 (2%)
Marital status:	Married	10 (83%)	38 (69%)
	Living with partner	2 (17%)	13 (24%)
	Single	0	4 (7%)

Data presented as median (interquartile range) and n (%)

The participants were aged between 30 and 43 years (median 36.5 years) and were from a range of ethnic backgrounds. The pregnancy experience that seven of the women described related to their first pregnancy / baby. For three of the women their pregnancy resulted in a perinatal loss (2 stillbirths and 1 early neonatal death). Of the nine women with surviving children, seven were born preterm, between 25 and 34 weeks of gestation*.* Eight babies were delivered by caesarean section; the remainder were vaginal deliveries.

The interviews ranged from 16 min, 50 s to 38 min, 30 s long (median 26 min). All of the women answered all of the questions. The impact of issues discussed on the participants was monitored throughout [33]. Although discussing their experiences required some of the women to reflect on upsetting events, none of them wanted to pause or discontinue the interview. Four broad themes were identified, each of which incorporates a number of subthemes (Table 3). There was no evidence of participants’ views differing according to their personal or pregnancy characteristics, however this may have been because the sample was too small to reveal such differences.

**Table 3:** Broad themes and subthemes

Broad theme	Subthemes
Before joining the EVERREST Prospective Study	▪ Fertility and previous pregnancies ▪ Early pregnancy ▪ Finding out something was wrong ▪ Transfer of care
Participating in research	▪ Joining the EVERREST Prospective Study ▪ Being part of the study ▪ Involvement in other research
Information and support	▪ Information and support experiences involving other health care professionals ▪ Seeking information ▪ Support networks
Looking back and looking forwards	▪ Making sense of what happened ▪ Looking to the future

## Before joining the EVERREST prospective study

The interviews began with the women discussing events up to joining the EVERREST Prospective Study:

### Fertility and previous pregnancies

Five of the women had previous successful pregnancies which had gone smoothly with the babies born well and at, or near, term. Two women had previous unsuccessful attempts at in vitro fertilisation (IVF) and the pregnancy that caused them to join the EVERREST Prospective Study was an IVF pregnancy. In their discussion both women alluded to the emotional pressures associated with infertility treatment:

*So we conceived by IVF, it was our fifth round of IVF. It was quite a stressful journey to fall pregnant in the first place and we were over the moon when we had that positive test –.*

*07*

Other women referred to their own health problems and their expectation that this may impact on their fertility and any pregnancy. In conjunction with this part of the discussion, they also described finding out that they were pregnant.

*I do have diabetes so I’d been seeing my GP about my medication and I talked to him about getting pregnant. The GP referred me to X ((hospital)) and by the time I had that appointment, I found out that I was pregnant. That was such a shock ((laughs)) – 09.*

### Early pregnancy

Before joining the EVERREST Prospective Study many of the women had experienced the symptoms commonly associated with early pregnancy. They had the usual antenatal screening and most were not unduly concerned about either their own health or that of their baby.

*I had a little bit of evening sickness rather than morning sickness but nothing irregular –.*

*04*

### Finding out something was wrong

The women continued by describing when problems in the pregnancy were identified. They explained what happened, how they were told and their reaction to the information. For some women confirmation of their baby’s growth restriction coincided with the onset of pre-eclampsia, vaginal bleeding or the identification of poor placental / uterine blood flow. For others, it was identified that their baby was smaller than expected during routine ultrasound scanning.

*Everything was going really smoothly until our 20 week scan … … … … they said that the baby was curled up in a ball so they couldn’t do the measurements but they said that happens. So we weren’t so worried at that point and then a couple of weeks later the same thing was happening but the measurement that they could take they said that the baby was a bit small and they referred us to fetal medicine – 07.*

All of the women talked about who first told them about the FGR and how they were told. Whilst most could not remember their name, they were able to describe their role. The women could all clearly recall their reaction to the news that their baby was smaller than expected. They described feeling ‘devastated’, ‘worried’, ‘upset’, ‘floored’ and ‘shocked’. One said she felt cursed, another said the news came as a ‘bomb-shell’.

*Oh devastated … … Absolutely devastated. You know, because we’d so much obviously longed to have the child and that. And then to be told, you know, it’s, so it’s almost, like, you know, you’re thinking, you know, if someone’s put a curse on you or something. Like, you’ve been through so much actually to get pregnant in the first place – 01.*

*Then I think, I don’t remember when, 24 weeks or so, one of the consultants said his liver blood flow was not right. So then it was even worse because he said that my baby might die at any time … … …………… … Yes, that was very stressful because at that point I was feeling his kicks and yes, that was really bad. I had to take some time off work. I couldn’t tell it to anyone, these things – 03.*

Some women experienced a more delayed response because they could not initially take in what they had been told. Others were more accepting or did not at first fully appreciate the severity of the situation.

*I think it took us probably until the next day really to really realise how upset we were. I think our initial reaction was acceptance and right ok and probably a bit shocked and then trying to work out what it meant really, which I think took some time and perhaps we didn’t really work it out until she was born. But we certainly took a bit of time, yeah – 04.*

Despite feeling worried and concerned by the news, most women appreciated being given frank, realistic and honest information in a sympathetic manner. They were also grateful that their baby’s problems had been picked up early in the pregnancy.

*I just thought, ok, well, I felt grateful that I was being referred to X ((hospital)) because I thought, oh, good, I’m going to a specialist place, I kind of felt lucky that it had been picked up – 08.*

Conversely a few women described being given the news in a way that could be deemed less supportive.

*Did he say that to you in those words? ((that the baby might die at any time)) – MH.*

*Yes, at the scan. It was like not really oh, just telling me the fact that, he may die, what can we do? I understand, you know, a doctor but they could have been a bit softer – 03.*

Some of the women were on their own when they were told of their baby’s growth restriction. This was usually because they had been asked to attend a second scan and were unaware of the reason for this. The impact of being given the news on their own was for some women compounded by a lengthy journey home afterwards. As a consequence, they made sure that they were accompanied by a family member or friend to all future scans.

*I was on my own. It was really, really hard – 11.*

*It was just everything as normal, until the 20-week scan in which case they obviously flagged it up as a bit small, and they didn’t tell me, they just asked me to come back, that was my hospital, asked me to come back the next week. And then I came back the next week on my own just thinking it’s because they hadn’t taken enough measurements or something, and then that I was told, oh, it was small. So unfortunately I didn’t come back with anybody because I thought it was just collecting some more data, but that was obviously quite a shock, that they didn’t let me know why they were asking me back, because I was on my own then – 08.*

### Transfer of care

Whilst a few of the women were under the care of the study site throughout their pregnancy the remainder were transferred there when the severe early-onset FGR was identified. These referrals reinforced the seriousness of the situation. However, they were reassured by the reputation of the study site and felt they would receive optimal care from specialist staff who had access to better facilities. They also believed they would be more likely to have a live baby.

*… … I think, I don’t know, probably realised the severity of it. So we were obviously, then I know, I’ve heard of, I know X ((hospital)) and that. But on the other hand, like, then I thought, actually well I’m probably in the best place, because they’ve probably got a bit more specialist staff there as well – 01.*

*I think, so a mixed reaction. I think it was a, oh my gosh, what is going on? What’s happening? It must be something bad if I’m being transferred there, but at the same time it’s like a, I’m going to the best place where they know a lot, so it should be okay – 02.*

## Participating in research

During the interview, the women talked extensively about being involved in research generally and the EVERREST Prospective Study specifically.

### Joining the study

The women often could not recall who specifically had recruited them to the EVERREST Prospective Study. However, they all remembered being told that taking part may help other families in the future. When deciding whether to participate, some drew on their own knowledge of research. Whilst some decided very quickly, others needed more time and this often included discussing the study with family and friends. None of participants felt pressurised to take part.

*Yeah, it was X ((member of research team)), she was so lovely. She was really, ‘cause she was really helpful and supportive all the way through … … … It was an instant decision. It was just sort of, it was just something that we thought if it’s going to help somebody else, then we’re happy to do it – 02.*

*I thought it was a good idea, I thought they might find a cure … ..I thought about it for a couple of minutes – 11.*

### Being part of the study

The women described the positive aspects of the way the EVERREST Prospective Study was conducted. Research team members were described as being ‘understanding’, ‘kind’, ‘supportive’, ‘helpful’, ‘sympathetic’ and ‘accommodating’. When giving examples of positive interactions some health professionals were specifically named. It was important to the women that the remembered their name and were ‘upbeat’ in their approach.

*I’d have to say I think both my husband and I feel that we were extremely well treated, the care we were given both by the fetal medicine team, perhaps even the fetal medicine team in particular and the EVERREST study was very, very good. I think we both felt it was done with a good degree of care and we’re very grateful to the people involved – 03.*

Whilst a few women said there was no direct advantage to them being part in the EVERREST Prospective Study, most thought there were a range of benefits. They felt they had received better care, additional and more detailed monitoring and prompt referrals. Being part of the research therefore enabled them to learn more about their baby. They also felt they had more opportunity to ask questions and felt this was empowering. Being part of the study also helped them to feel less isolated and that they were contributing to something important.

*That is one thing to add that I think the fact that you know, that during that time, that there is some medical research taking place, that people are working on it does give some comfort I think. Perhaps it does make you feel less isolated at points as well, that it’s being looked at globally essentially, it’s across countries. Perhaps you also do feel less afflicted and perhaps there is that element to it – 03.*

It was apparent that most of the women could not distinguish between fetal medicine staff and research team members and where their roles overlapped. Similarly, most women were unclear what the usual care pathway was when a growth restricted pregnancy was confirmed and the specific elements of the EVERREST Prospective Study. A few women acknowledged their uncertainty about this.

*I believe that X ((doctor)) and her team saved my baby’s life, the decisions they made when they decided when to give me the steroids and when it was best for her to be born. But I don’t know if their decision making was related to the extra information they gathered as part of EVERREST or whether they would have made those same decisions regardless of me being part of EVERREST – 07.*

Some women identified other more peripheral benefits of taking part in the research. This included being able to take a day off work to attend appointments, being able to take family members with them, combining appointments with social events and the opportunity to get involved with other related activities.

*I did a ((radio)) programme with them ((the research team)) as well, you know, they were talking about growth restriction. … …*. I *got involved with the programme – 01.*

Negative factors associated with being part of the EVERREST Prospective Study mostly related to logistical challenges such as getting time off work, the cost of additional travel to appointments and arranging child care. However, these negative issues were felt to be minimal and were outweighed by positive aspects.

*The travelling was tiring but I always came back calm and happy – 06.*

The women talked extensively about why they took part in the research. Initially they gave altruistic reasons saying that they wanted to help to improve care for other families. They also felt it was a way of ‘giving something back’ for the care they had received. One mother felt she was an unusual case and so had something specific to offer the research.

*Happy to take part in anything that’s going to help anybody else further along the line, so I had no problems with doing it or taking part in anything at all. If it benefits somebody else, very happy to – 02.*

As the discussion continued, the women talked about more personal benefits that influenced their decision to participate. They thought they would have more information and greater reassurance. They also thought they would receive better care, even though within the recruitment process it was made clear that this was would not be the case.

*I guess we probably made the decision thinking if we join the study that we might get better care, I know they always say that’s not the case, the care will be the same. But all of those things were said to us so it didn’t help, it didn’t stop me thinking that we might get better care – 07.*

The women were asked if they had understood at recruitment what participation would involve. Most said this had been as they expected. Whilst a few felt that they had not fully understood, this did not concern them and they were pleased to have taken part.

*There haven’t been any surprises. I probably didn’t fully understand what was involved at the time, just because I’m a first time mother and was involved in the pregnancy, so at the time I just thought if it helps X ((hospital)), I’ll help – 05.*

The women were asked if there was anything that the research team could have done differently. Whilst most did not identify any changes, a few made some suggestions. These included more face-to-face discussion rather than the provision of written information at recruitment, more updates and guidance on accessing information about growth restriction.

*I did a lot of research online about small babies and that kind of thing … … … .. I think I would have liked to have had or been given, or been pointed in the direction of credible studies and ones that were well thought of, rather than me having to try and find them myself – 08.*

### Involvement in other research

Although the focus of the interview was the EVERREST Prospective Study, a few women talked about being involved in other research not related to the EVERREST Project. They described reaching a threshold when asked about their or their baby’s potential participation in other studies. In declining other research, they felt the need to protect their baby, particularly if their child was preterm. They also indicated that research teams should be sensitive when approaching families about studies at a difficult time or when they were already taking part in other research.

*I think by the time she came to the end of her time in the neonatal unit we’d been asked to join a couple of other additional studies and I have to say I did get to the point where I started saying no. Because at that stage I felt here’s this tiny baby we were desperately trying to get her home and I felt she’d had enough. And actually I felt we’d had enough … … I think we reached the point by the end of that, what was nearly four months where we just thought actually we want to protect our child and we feel like we’ve given enough. I think the EVERREST was not a problem – 04.*

The women also talked about the likelihood of getting involved in other research in the future. They felt their understanding of the importance of research and their positive experience with the EVERREST Prospective Study meant they would at least consider taking part in other studies.

*I suppose I would judge each study as it came along and see what happened, but most of the time I’d be happy to take part in mostly whatever it is that’s being offered – 02.*

As part of this discussion the women talked about the likelihood of them agreeing to be involved in the EVERREST Clinical Trial. This would require them to consent to the possibility of having maternal uterine artery adenovirus VEGF gene therapy. A few women were aware of this planned research. Some women felt the question was too hypothetical and that they would only really know how they would respond when faced with the situation. Conversely, others felt confident that they would agree to participate. This was because the study would be building on the work of the EVERREST Prospective Study, by a team of researchers they knew and trusted.

*I think if I’ve already helped by taking part in the EVERREST study, I mean obviously the next.*

*stage for you guys is to be able to make a comparison … .. So I would be happy to help – 05.*

*For now I will say yes, because if there is anything, God forbid I’m in this situation again, anything to help my baby, while in there. I’ll be happy to have it because at the end of the day … … if there’s anything to help her, yeah, I’d be happy with that – 10.*

Other women were more uncertain because of the unknown impact on the baby or the study outcome. They also felt that they would not want to jeopardise what potentially for them would already be an ‘at risk’ pregnancy.

*Like I said, I don’t want to take anything that could harm by baby, my baby was small because of my diabetes and all that – 09.*

*Only if it wasn’t going to harm the baby – 06.*

Some of the women went on to say that they would only agree to take part if it was guaranteed that they would receive the study intervention (rather than a placebo or conventional treatment) or if the intervention had previously been tested on humans.

*I think if I was in that position then I would want to have the gene therapy, but I wouldn’t want to be offered a placebo, I would only want to know I was agreeing to have it or not to have it; and I think I probably would agree to have it – 08.*

## Information and support

Within the narratives, the ways in which health care professionals provided more general information and support was an important factor in shaping the women’s overall experiences. They also talked about proactively seeking information and support themselves.

### Information and support experiences involving other health care professionals

Some women gave mixed reports of information provided by health care professionals who were not part of the research team. In some cases they were concerned about an apparent lack of knowledge or being given contradictory information.

*I was just left kind of thoroughly unimpressed with her kind of knowledge of my, well, she hadn’t read any of my notes, and her responses were very, oh, we’ll do this, and we would do that, but only based on what I’d told them, not kind of based on her having looked at my notes and thought about it or anything like that, I felt. And that appointment kind of coincided with I think it was kind of, or me or someone saying about transferring my care, and so that kind of just cemented the decision – 08.*

However, the same participant also described a positive interaction she had with another health care professional.

*After every scan the head midwife ((at local hospital)) who originally referred me to X ((hospital)), she phoned me just to kind of catch up how I’d got on and that kind of thing. I thought that was really nice – 08.*

### Seeking information

In addition to the information from health professionals, most women had also accessed information from other sources. This included speaking to family and friends with specialist knowledge, consulting other health professionals and accessing information via the Internet. With regard to the latter, they said it was difficult to access appropriate and reliable information. Consequently, the information that they gained was not always helpful.

*I spoke to my aunt who is also a midwife and she started giving me scenarios of other parents and friends that she knows who have been given negative reports about their scan. By the end of the day, the baby came out perfectly well. But, you know, I just suppose she was trying to reassure me about what might happen – 10.*

*So that’s why I decided to go in the private as well to make sure, to have more than one answer, more than one, not because I don’t believe in NHS, but I just like to have more information as I can, and to see if I have any information like oh, your baby okay and your pregnancy’s fine – 12.*

### Support networks

The women described support networks that they accessed and the importance of their partner and their wider family. For one woman her church provided support whilst another found talking to a psychotherapist helpful.

*I have a family. Massive family network. My husband’s family and my family, as soon as they knew what had happened, my parents travelled down from X ((location)). X’s ((husband’s)) family in and around X ((location)), so we were, you know, there was, inundated with support. So in that sense, no there was a load of support – 01.*

## Looking back and looking forwards

As the discussion continued, the women reflected back on their experiences and also looked to the future.

### Making sense of what happened

The women talked about the birth and their baby’s subsequent care. They described the impact of their experiences on their health and emotional wellbeing as well as the impact on family members and particularly their other children. They also spoke about accepting what had happened and ‘moving on’.

The women were clearly able to recall events surrounding the birth of their baby. In most cases the delivery was prompted by a deterioration either in their own health or that of the baby. Although for many of them a premature birth had been anticipated, they nevertheless felt ‘shocked’, ‘anxious’ and ‘stressed’ when this actually occurred. One woman having been told her baby needed to be delivered early, encountered additional anxiety when the delivery was delayed due to a lack of neonatal intensive care resources.

*I think all the way along X ((doctor)) had said in this particular case she would feel uncomfortable going much beyond 28 weeks, because I think she felt it was a very fragile situation. So, nevertheless I still felt very shocked when someone turned round and said, right I think we’ll deliver this baby tonight – 04.*

Of the ten live born babies, eight required neonatal unit (NNU) care, five of whom were cared for in more than one NNU. One baby died at 9 days of age. The women described their baby’s time in the NNU as a ‘rollercoaster’. They talked of their distress on being separated from their baby and being surrounded by heathy babies on the postnatal ward. Some women valued the extra time in hospital that they were able to spend with their baby, whilst others thought they had been discharged home too soon. Women whose baby was cared for in more than one NNU had mixed feelings about this. Some were initially, and remained reluctant about their baby being transferred, whilst others soon adapted to the new environment.

*What was bad was after I had been discharged from X ((hospital)) and my daughter was still there, going every day with my caesarean – 10.*

*I didn’t like the idea of that ((baby transferred to another NNU)) at all. I knew it had to happen because X ((hospital)) is very much high dependency, and X ((hospital)) was nearer home, and I was travelling in every day, but it’s quite, it’s, as for any mum, it was quite traumatic –.*

*05*

The women went on to reflect on to their own health after the birth. They talked about recovering from a caesarean section, the ongoing management of their health problems and their emotional wellbeing after the birth.

*I suppose, you know, I just went through a difficult time mentally. And, you know, to be fair that’s not gone away. You know, then X ((baby)) was in hospital this year with pneumonia, no last year with pneumonia at Eastertime. And then I just find that now it’s just triggered any, so X ((baby)) what I went through with X ((baby)) has just triggered something in me that any, sort of, major change in my life or trauma, it gets me, you know, it’s gets me back to that state again and I’ve got to really be careful that, you know, at the time, recognise the signs early on and take the necessary actions really – 01.*

Some of the women reflected on the impact of their experiences on their other children. In the main, they felt events had a negative impact, particularly if the baby had died.

*I’ve tried to explain to him ((other son)) every day. Of course he feels like he’s supposed to have a baby brother, everybody’s got a brother or sister and I’ve not. It’s like that. But I just explain to him I had operation so they took it ((the baby)) out from my uterus, my uterus is new, so in one year time I can have another baby if I desire. So it’s something like he can have a hope. If God gives me this opportunity I will have another baby in the future – 12.*

Within their reflection, the women discussed their understanding of the reasons for the growth restriction. Some had been told this was due to placental insufficiency, chromosomal anomalies or pre-eclampsia. Others said they had not been given a reason and surmised the likely causes. One mother wondered if the impact of invasive procedures during her pregnancy was a possible cause.

*I still don’t know why, I was worried at first that I’d picked something up in Africa – 06.*

When reflecting on their experiences, for some women their religious faith or belief in fate brought comfort and acceptance. Others felt ‘lucky’ and ‘grateful’. They also felt something positive had come out of their experience because they had been able to contribute to the EVERREST Prospective Study. Several women felt it was now a time for them to ‘move on’. One woman whose baby died in utero felt she had to ‘keep on going’ for her other child.

*I think, you know, I was one of the lucky ones and we’ve come out of it the other side. And I’m sure there are stories where, you know, it’s not been such a happy ending. You know, so I just think, do you know what, it’s what’s meant to be is meant to be. And what your destiny will be, isn’t it? And so that, you know, that, it’s one of those things, just a part of life, isn’t it, unfortunately. So sometimes, you do question why you? Because you went through so much to get there in the first place. But, you know, again you’ve just got to, now looking back you’re just grateful for where she is now – 01.*

### Looking to the future

In looking forwards, some women considered the possibility of having another baby. All those who thought they might, felt reassured that they would be closely monitored.

*I went there ((hospital)) last week, they gave me some information about if I get pregnant again – 11.*

One of the women who had previously experienced a neonatal death was pregnant at the time of the interview. She expressed her anxiety about the outcome even though she had been reassured that all was well:

*Emotionally it’s sort of all mixed because of my last pregnancy, and the little one not making it. I know that this one is fine and there are no issues, hopefully, so far, but there’s always like that underlying anxiety – 02.*

The women also considered the health of their baby now and their baby’s likely health in the future. Some babies had ongoing health problems which usually related to prematurity. All babies continued to be small. Whilst some women remained concerned about this, others were more sanguine. Part of the discussion in relation to this focused on their child’s current weight and possible weight gain in the future.

*He was putting weight on really well ((in the NNU)), he went up from 0.4 centile to second centile quite quickly. The thing is though, he’s quite funny with food. If he’s not in the mood or the teeth are hurting, he won’t eat. But in the nursery, he eats everything. I wouldn’t say there was something wrong with him physically. It’s just he’s been playing with me – 03.*

## Discussion

The qualitative approach taken in this study was an appropriate way in which to learn about the experiences of women of having a pregnancy complicated by growth restriction and of EVERREST Prospective Study participation. Conducting the interviews by telephone was an effective and practical way of exploring the women’s experiences [25, 34]. This interview study provides valuable insights to the emotional impact of having a growth restricted pregnancy, the challenges the women faced when their care was transferred, their information and support needs, their perceptions of the care they received and their experiences of being part of a research study. The women spoke frankly and whilst acknowledging the distress and despair they felt at times, they also seemed to enjoy talking about what happened to them. The experience of having a growth restricted pregnancy remained very real and for some had a lasting psychological impact. The women talked openly about their worries, concerns and fears when the growth restriction was confirmed and particularly about the likelihood of their child surviving or of preterm birth. The women often referred back to the exceptional care they had received. Those whose experiences resulted in a stillbirth or neonatal death seemed to appreciate the opportunity to talk about their baby.

The women talked in detail about why they had agreed to take part in the EVERREST Prospective Study. In common with the findings of other similar studies they mainly gave altruistic reasons for their participation [35–37]. They often commented on ‘giving something back’ and were keen to influence the future provision of care for women and families facing a similar situation. In making the decision to participate, they felt the study was unlikely to impact on either them or their baby in a negative way. Some women also talked about the personal benefit to them and particularly having access to more information and support and feeling less isolated at a difficult time. They generally assumed they would receive better care [38, 39]. Although in some cases participation presented additional obstacles [35] such as travel and organising child care, all of the women were pleased to have taken part.

There were a number of aspects of the EVERREST Prospective Study that the women valued which maintained their engagement. This included the way in which they were recruited, ongoing contact with the research team, feeling that they were part of something important and reducing their feelings of isolation [35]. The more negative encounters experienced by some of the women with other health care professionals, reinforced their trust in the EVERREST Prospective Study research team.

Issues surrounding information giving and information retrieval were a large component of the interviews. This is unsurprising given that pregnancy, childbirth and the early postnatal periods are key times when women and their partners seek information, particularly if they are first-time parents [40, 41]. For the women in this study, the growth restriction, potential preterm birth and concern about their baby’s survival heightened their need for information. The ways in which information was initially and subsequently given about the growth restriction were key aspects of the women’s experiences. As has been identified in other studies of clinician-service user communication, the women had differing information needs [42–44]. Whilst they generally preferred frank information given in a direct way, a few preferred a softer approach. This indicates that information-giving requires a flexible approach from health care professionals in order to ensure that the strategies adopted suit the needs and wishes of individual woman and their families [44, 45].

Two interlinked issues relating to communication and information-giving at the diagnosis of severe early-onset FGR were pertinent to the narratives of some of the women. The first was not understanding why they had been asked to come back to the hospital for a repeat scan. As a consequence some women returned for this follow-up appointment on their own. The second was being given distressing information when they were on their own and the added impact of having to travel home alone afterwards. The health care professionals may have not wanted to unduly or unnecessarily alarm the women about the reasons for the follow-up scan. Nevertheless, these issues highlight the need for health care professionals to carefully consider what information they give about follow-up appointments and the value of women being be accompanied to such appointments.

The internet was used as an information source by a number of the women and their partners to clarify information they had not fully understood, to access more detailed information and to inform their decision-making [46, 47]. However, the limitations of using the internet as an information source were acknowledged and have previously been recognized [44, 48]. Family and friends were other useful information sources of information [40]. This is a factor for health care professionals to consider because this could be a source of contradictory information.

It is important to acknowledge factors that may have affected what was reported. None of the women had difficulty recalling what had happened. Indeed, interviewing participants at least one-year post-experience enabled them to reflect on their experiences as a whole rather than focusing solely on their immediate reactions. However, this meant for some women that the entirety of their experience may have been shaped by other factors such as having a preterm baby requiring neonatal intensive care. Nevertheless, diversity within the sample enabled a range of experiences to be described [49]. As in any study, it is only possible to reflect the opinions and experiences of women who agree to participate. It is possible that the views of women who did not take part in this interview study may have differed from the views of those who did.

## Conclusions

This qualitative study provides insight to women’s experiences of having a growth restricted pregnancy. Their retrospective reports and reflections illustrate their recall and understanding of what had happened, the impact of the care and support they had received and their experiences of being part of the EVERREST Prospective Study. The interviews also facilitate understanding of women’s perceptions of care and highlights aspects of care delivery and being part of a research study that were important to them. Interviewing women at this time point, when they have had time to reflect on earlier events, resulted in a more balanced and thoughtful view of their experience. A strength of this qualitative work is that it goes beyond the women’s immediate reactions to their experiences.

## Additional file

Additional file 1: EVERREST interview study – topic guide. The topic guide used to conduct the semi-structured interviews (DOCX 18 kb)

## Abbreviations

CS: Caesarean section
FGR: Fetal growth restriction
G: Gravida
IVF: In vitro fertilisation
NNU: Neonatal unit
P: Para
VEGF: Vascular endothelial growth factor
