# Supplementary material for: Pregnant women’s experiences and perceptions of participating in the EVERREST prospective study; a qualitative study
Source: BMC Pregnancy Childbirth. 2019 Apr 30;19:144. doi: 10.1186/s12884-019-2277-8 (PMC6492343; doi:10.1186/s12884-019-2277-8)
Supplement: Supplementary file 1 — EVERREST interview study – topic guide. The topic guide used to conduct the semi-structured interviews (DOCX 18 kb) [file 12884_2019_2277_MOESM1_ESM.docx]

EVERREST INTERVIEW STUDY – TOPIC GUIDE

Thank you for agreeing to take part in this interview. Before we begin, I would like to clarify some information with you. With your permission, the interview will be recorded. There are no ‘right’ or ‘wrong’ answers to the questions I will ask you, I am interested in your opinions and experiences. I will take out the names of anyone you mention during the interview to protect their identity. If there are any questions that you do not wish to answer, please say and we will move to the next question. If you wish to pause or stop the interview at any time, again, please let me know. Are there any questions that you would like to ask me about the interview before we begin?

Are you happy to proceed? And for the interview to be recorded?

**The following instructions will not be read out to the participant: the topic guide consists of key questions / subject areas that will be explored during the interview. During the interview the Interviewer will ensure all questions / subject areas are addressed. Follow-up or probing questions (indicated in italics) will be used as appropriate in accordance with participant responses. The structure of each interview will therefore be determined by participant responses. Consequently no two interviews will be conducted in exactly the same way.**

Thank you for agreeing to take part in this interview.

I would like to hear about your experiences of taking part in the EVERREST Prospective Study. However, can we start by you telling me about your pregnancy experience up to the point that you joined the study?

*When identified baby was small*

*Other pregnancy related problems*

*Antenatal care including screening*

*Own perception of problems*

*Previous pregnancy experiences*

Can you tell me about your response to it being confirmed your baby was small

*How told and who by*

*Expected / unexpected*

*Reaction / impact*

Can you tell me about when you joined the study?

*When / how the study was introduced to you and by whom*

*Initial response to the idea of joining the study*

*Making the decision to join the study: discussed with anyone else, difficult / easy decision, influencing factors*

Can you tell me about your experiences of taking part in the EVERREST Prospective Study?

*Benefits / advantages / positive experience: psychological, practical, logistical*

*Drawbacks / disadvantages / negative: psychological, practical, logistical*

*How feel about it now*

*Anything unexpected / surprising*

*Looking back, did you understand what was involved?*

What impact has this experience had on the likelihood of you participating in research in the future?

*More / less likely*

*Why*

*Any research / specific types of research*

The aim of the proposed EVERREST project is to trial the use of a growth factor therapy which is given to the mother to try to help her baby grow. This has improved fetal growth in animals but is not yet available for humans. What would be your response to being asked to take part in a study like that?

*Growth factor not previously used in humans*

*Implication of randomisation*

*Factors that would help make decision*

Looking back is there anything the research team could have done differently?

*Information about the study*

*Way study conducted*

*Contact / interaction with research team*

Looking forward, how are you now?

*How is baby (if appropriate)*

*Impact of taking part in study*

Is there anything else about being part of the EVERREST Prospective Study that we haven’t discussed that you would like to tell me about?

Thank you for your time today. I am sure what you have told me today will be very helpful to the study. Just to remind you, any names you have used will not be included when we analyse the interview.

**Reminder prompt will be used as appropriate: if following the interview you feel you want to talk to someone about the issues we discussed you can contact your GP or Health Visitor. You may also find it useful to contact the following charities: ARC (Antenatal Results and Choices) 0845 077 2290; BLISS 0500 618140; SANDS 020 7436 5881 – see participant information leaflet.**
